# Differential Expression and Prognostic Significance of STARD3 Gene in Breast Carcinoma

**DOI:** 10.22088/IJMCM.BUMS.10.1.34

**Published:** 2021-05-22

**Authors:** Abdul Fattah Salah Fararjeh, Ali Al Khader, Ezidin Kaddumi, Maher Obeidat, O'la AL-Fawares

**Affiliations:** 1 *Department of Medical Laboratory Analysis, Faculty of Science, Al-Balqa Applied University, Al-salt, Jordan.*; 2 *Department of Pathology and Forensic Medicine, Faculty of Medicine, Al-Balqa Applied University, Al-salt, Jordan.*; 3 *Department of Pathology, Al-Hussein Salt Hospital, Al-salt, Jordan.*; 4 *Department of Basic Medical Sciences, Faculty of Medicine, Al-Balqa Applied University, Al-salt, Jordan.*

**Keywords:** Breast neoplasm, STARD3, HER2, prognosis, computational biology

## Abstract

StAR related lipid transfer domain containing 3 (*STARD3*) gene has been reported to be co-amplified with human epidermal growth factor receptor 2 (*HER2*) in breast carcinoma. STARD3 is necessary for cholesterol transfer and metabolism in tumor cells. The possible role played by *STARD3* as a diagnostic and prognostic biomarker was investigated in breast cancer (BC). Data mining was performed using several bioinformatics websites to investigate the correlation of *STARD3* with BC and its molecular subtypes, and conventional PCR was used to detect the *STARD3* mRNA levels in a panel of BC cell lines. *STARD3* was overexpressed in BC more than the other types of cancer. The results also showed that *STARD3* expression was significantly associated with HER2^+^ BC tumors and BC cell lines, and low STARD3 mRNA and protein expression levels were observed in estrogen receptor-positive (ER^+^) and triple-negative BC (TNBC) patients. Moreover, high *STARD3* expression levels predicted worse overall survival (OS), relapse-free survival (RFS) and disease metastasis-free survival (DMFS) in BC, and HER2^+^ BC. Notably, low expression of *STARD3* was associated with poor OS in ER^+^ BC. Our findings suggest that *STARD3* may have strong diagnostic and prognostic value for HER2^+^ breast carcinoma.

Breast cancer (BC), which accounts for nearly 30% of new cancer cases among females worldwide, is the most common type of cancer in this population (1). Importantly, BC is the most common cause of cancer deaths among females between 20 and 60 years of age (2). BC is classified into 4 main molecular subtypes, with each having important prognostic and therapeutic associations: luminal A (estrogen (ER)-positive, human epidermal growth factor receptor 2 (HER2)-negative); luminal B (ER-positive, HER2-positive); HER2^+^ (ER-negative, HER2-positive); and triple negative breast cancer (TNBC) (ER-negative, HER2-negative, progesterone (PR)-negative) carcinoma (3). Luminal A (ER^+^, HER2^-^, and PR^-^) tumors have the most favorable outcome, partly due to their responsiveness to anti-hormone therapy (4). On the other hand, the HER2^+^ molecular subtype is associated with a worse prognosis and early metastases. The advent of targeted therapy has markedly improved the prognosis in HER2^+^ BC patients. However, these tumors still cause a persistently high number of deaths, and further biomarker studies are required to develop novel therapies (5, 6). Overexpression of the receptor tyrosine kinase HER2, caused by 17q12-q21 amplification, occurs in approximately one-fifth of all BCs (7). HER2 is an epidermal growth factor receptor that plays a key role in BC development through the activation of downstream signaling pathways, such as RAS/MAPK and PI3K/AKT, which regulate the survival and proliferation of tumor cells (8). However, HER2-independent signaling pathways have been implicated in cancer progression and in the aggressive behavior of these tumors, which might explain the failure of HER2-targeted therapy in a significant number of patients (9, 10). Several genes have been reported to be co-amplified with *HER2* (11), and StAR related lipid transfer domain containing 3 (*STARD3*) is one of these genes. *STARD3* has been suggested to contribute to the proliferation of HER2^+^ cell lines. The *STARD3* gene encodes a cholesterol-binding membrane protein (12, 13). This protein localizes to late endosomal organelles (LEs) and has an N-terminal domain that targets the protein to the LE membrane and a C-terminal domain with a cytoplasmic cholesterol-binding site. *STARD3* may play roles in the actin-dependent movement of LEs and cholesterol transfer between LEs and other membrane-bound organelles, such as mitochondria (14, 15). In BC, cholesterol metabolism has been shown to be dysregulated, as measured by the levels of cholesterol or its metabolites (16, 17). *STARD3* overexpression was reported to enhance cholesterol biosynthesis by increasing the expression of cholesterol synthesis enzymes (18). In this study, we analyzed *STARD3* mRNA expression levels in various BC and normal breast cell lines. Moreover, we performed a web-based evaluation correlating *STARD3* with BC molecular subtype (i.e., HER2 status, ER status and TNBC status) and patient survival.

## Materials and methods

**Cell lines**

Seven BC cell lines and 1 normal breast cell line were purchased from American Type Culture Collection (ATCC, Manassas, VA, USA), and were used in this study (19). Of these cell lines, two were ER^+^ (MCF7, T47D, and BT-474), three were HER2^+^ (BT-474, SKBR3, and HCC1419), two were triple-negative (MDA-MB-231 and MDA-MB-436) and one was normal (MCF 10A). All BC cell lines were provided by Professor Ho Yuan Soon Laboratory, Taipei Medical University, Taiwan, and were cultured with suitable supplements according to the recommended conditions.

**Conventional qualitative PCR**

Using TRIzol reagent (Invitrogen, Carlsbad, CA, USA), total RNA of BC and normal breast cell lines were extracted according to the protocol provided by the manufacturer. The *STARD3*-specific forward 5’-TCCCCATCGTCTCTTTT GTC -3’ and reverse 5’-CGCTCCTGAGCAGAGA AACT-3’ primers were used. For the* GAPDH* gene, the specific forward 5’- TGAAGGTCGGAGTC AACGGATTTGGT -3’ and reverse primers 5’- CAT GTG GGCCATGA GGTCCACCAC-3’ were used and purchased from Genomic Company, Taipei, Taiwan (https://en. genomics.com.tw/ about). PCR was used under the following conditions: 94°C for 4 min, denaturation at 94°C for 30 s, annealing at 55°C for 30 s, and extension at 72 °C for 30 s. The cycles were repeated 33 times. The experiment was repeated two times.

**Expression profile of** ***STARD3*** **in human cancers**

GEPIA, gene expression profile integrative analysis, and Oncomine bioinformatic tools were used to analyze the expression pattern of the* STARD3* gene at the mRNA level in normal vs tumor tissues in BC and other types of cancers. GEPIA is a web-based analysis tool that provides expression data at the RNA level from TCGA and GTEx projects (http://gepia.cancer-pku.cn/index. html), which contains more than 9,700 tumors and 8,587 normal samples. However, the Oncomine database (http://www.oncomine.org) contains more than 700 datasets in different types of cancer with high-quality and large study cohorts (20). 

**Receiver operating characteristic** **(ROC) plotter for the sensitivity and specificity of** ***STARD3***

ROC plotter (http://www.rocplot.org/) was able to predict the association between gene expression and response to therapy using the transcriptome level of more than 3000 BC patients. ROC plotter is the first online database used to validate the predictive biomarkers.

**Analysis of** **STARD3** **protein expression using the Expression Atlas**

The Expression Atlas (https://www.ebi.ac.uk/ gxa/home) is a database tool used for STARD3 expression level evaluation based on proteome analysis. The Expression Atlas is a powerful tool to provide information about gene and protein expression. This tool is involved in data curation and analysis, and is open access for science resources.

**Breast cancer gene expression mining tool (BC- genExminer v4.5)**

BC-GenExMiner is a bioinformatic mining tool that uses more than 10,500 DNA microarray findings and approximately 4700 RNA-seq transcriptomic findings for BC (http://bcgenex. Cen tregauducheau.fr/BC-GEM/GEM-Accueil. php?js= 1). This tool was used to study the expression of *STARD3* in BC subtypes.

**Kaplan-Meier plotter**

The prognostic significance of *STARD3* was assessed using the Kaplan-Meier plotter web-based tool (http://kmplot.com/analysis/). The patients were divided into two groups, high and low, based on the median expression of *STARD3 *at the mRNA level, and survival analyses were performed without follow-up restrictions. To determine the prognostic value of* STARD3*, we selected BC patients and chose the *STARD3* gene. Next, the cutoff value and the selected overall survival (OS), relapse free survival (RFS) or progression free survival (PFS) were automatically calculated.

**Statistical analysis**

Data analysis was conducted by Student t-test for analyzing the association of *STARD3 *between ER^+^, TNBC cell lines vs HER2^+^ cell lines. Statistical significance was defined as P <0.001. All data were expressed as mean ± SD. 

## Results

***STARD3*** **is more highly expressed in breast cancer than in other types of cancer**

**Fig. 1.**
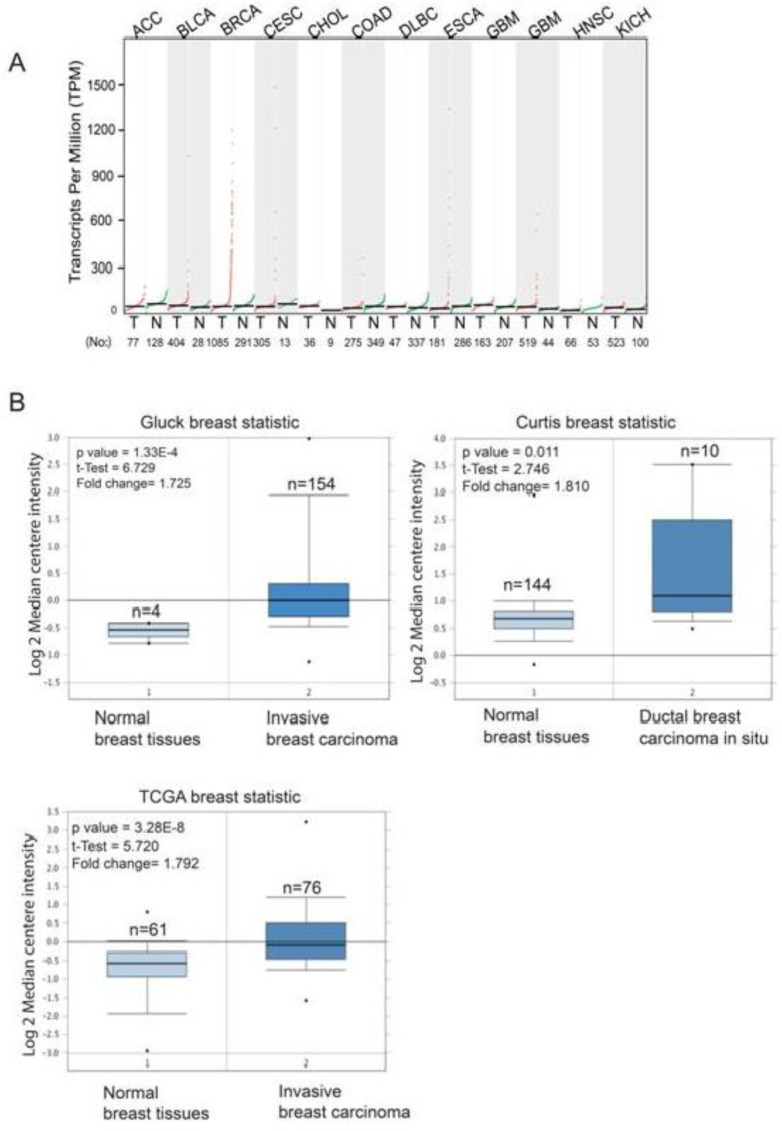
**Transcription level of** ***STARD3*** **in different types of cancers. **A (The graph was generated from the GEPIA website, which indicates the expression of *STARD3* at the mRNA level in several types of cancers. The red dots indicate the expression of *STARD3* in tumor tissues, and the green dots indicate the expression of *STARD3* in normal tissues; B (Box plots were derived from the Oncomine database from the Gluck breast statistic, Curtis breast statistic and TCGA breast statistic, which indicate the expression level of *STARD3* in normal (left) vs tumor (right).

The distribution of *STARD3* expression in different types of human cancers (n =33) in comparison to adjacent normal tissues was obtained from the GEPIA web-based RNA expression tool (Figure 1). The highest expression of *STARD3* was observed in BC compared with other types of human cancer. In addition, *STARD3* was overexpressed in breast tumor tissues compared with adjacent normal breast tissues.

We subsequently used the Oncomine database to compare the expression of *STARD3* in cancer tissues with that in normal tissues. The results demonstrated that *STARD3* is overexpressed in BC in comparison with normal breast tissues in most studied datasets (Figure 1). In Gluck’s dataset, *STARD3* was significantly overexpressed in invasive breast carcinoma compared to normal breast tissues (P = 1.33E^-4^) (Figure 1B). Additionally, the expression of *STARD3* was significantly higher (P = 0.011) in ductal breast carcinoma *in situ* than in normal breast carcinoma according to Curtis breast statistics (Figure 1B). According to TCGA breast statistics, *STARD3* was also overexpressed in BC compared with normal tissues (P = 3.28E^-4^) (Figure 1B).

**Fig. 2:**
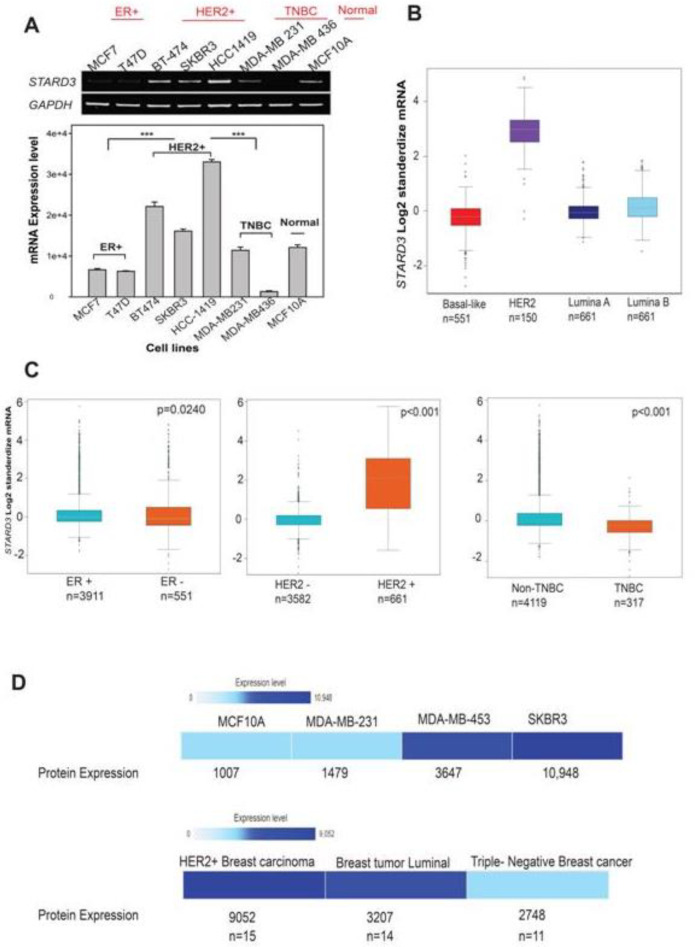
**Differential expression of** ***STARD3*** **in breast cancer.** A (Detection of *STARD3* mRNA levels in cancerous cells (MCF7, T47D, SKBR3, BT474, HCC1419, MDA MB231, and MDA-MB436) and normal cells (MCF10A); B&C (The correlation between *STARD3* expression and molecular subtypes (ER, HER2, PR, and TNBC statuses) in BC. The data were derived from BC gene expression minor (BC- genExminer v4.5); D (Expression atlas heat map indicating the protein expression of STARD3 in several cell lines (MCF10A, MDA-MB-231, MDA-MB-453 and SKBR3), and in clinical samples (HER2^+^, BC luminal A, and TNBC).

**Fig. 3:**
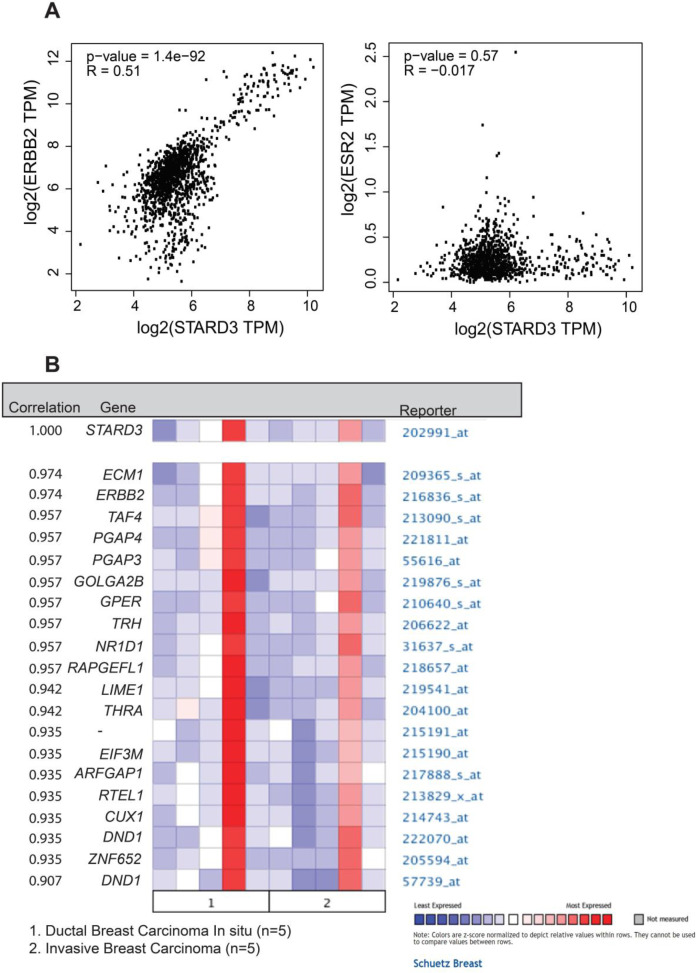
**Analysis of the association between STARD3 and HER2.** A (The dot plot graph represents the correlation between STARD3 and HER2 (left) and STARD3 and ESR2 (right), which were obtained from the GEPIA website; B (The graph indicates the genes most correlated with STARD3 in ductal and invasive breast carcinoma derived from the Oncomine database

**Fig. 4.**
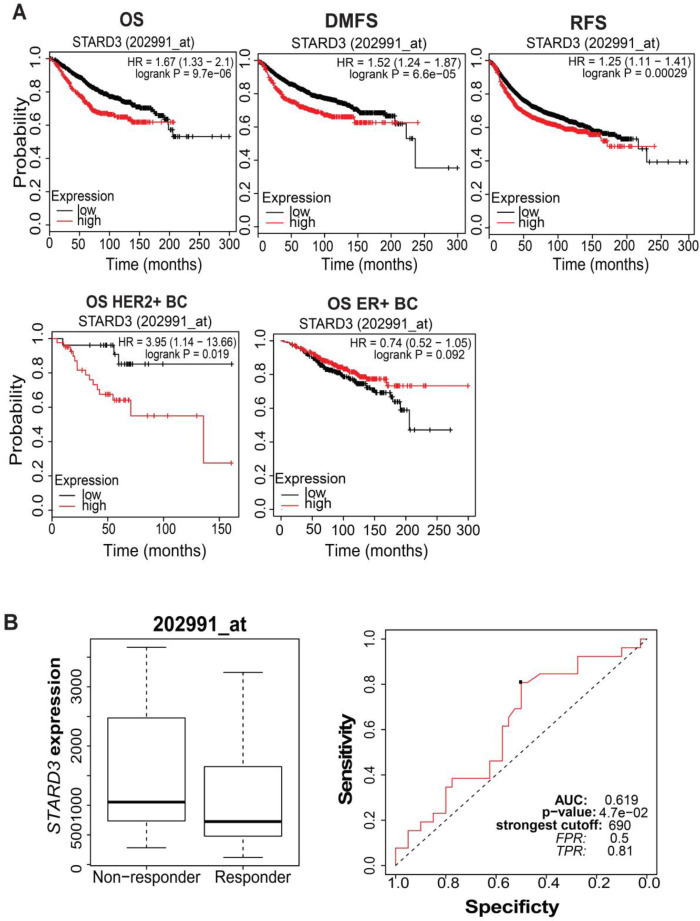
**Prognostic significance of STARD3 in breast cancer.** A) The prognostic significance of STARD3 in BC based on overall survival (OS), relapse-free survival (RFS), and disease metastasis-free survival (DMFS) based on the Kaplan-Meier plotter website; B) OS for HER2+ and ER+ BC patients based on high vs low STARD3 mRNA expression; C: ROC curve analysis shows the sensitivity and specificity of STARD3 in predicting the patient response to treatment (trastuzumab).

***STARD3*** **expression was associated with** **HER2**^+^** breast cancer**

To examine the mRNA levels of *STARD3* among BC subtypes, namely, luminal A, luminal B, HER2^+^ and TNBC, we used several BC cell lines: ER^+^ (MCF7, T47D, and BT-474), HER2^+^ (BT-474, SKBR3, and HCC1419), triple-negative (MDA-MB-231 and MDA-MB-436), and a normal cell line (MCF 10A) (Figure 2A). The mRNA expression of *STARD3 *in HER2^+^ cell lines BT474, SKBR3, and HCC1419 was significantly higher than that in ER^+^, TNBC, and normal cell lines (Figure 2A). In addition, based on the Box and Whisker RNA-seq database, high expression of *STARD3* was found in HER2^+^ patients (n = 150) in comparison with the other BC subtypes (P <0.0001) (Figure 2B). To confirm these results, we analyzed *STARD3* expression in BC subtypes (ER^+^ vs ER^-^); (PR^- ^vs PgR^+^); (HER2^+^ vs HER2^-^) and (TNBC vs non-TNBC) using BC-GenExMiner v4.5. According to the breast statistics dataset, the *STARD3 *level was higher in HER2^+^ than HER2- cells (P < 0.001), higher in ER^-^ than ER^+^ cells (P <0.001), higher in PR^-^ than PR^+^ cells (P <0.001), and higher in non-TNBC than TNBC cells (Figure 2C). We also used the Expression Atlas website to analyze the protein expression datasets of *STARD3* in BC cell lines and in clinical samples. In keeping with our results, *STARD3* expression was determined to be higher in HER2^+^-overexpressing MDA-MB 453 cells than in MDA-MB-231 TNBC cells and MCF10A normal MDA-MB-231 cells. In addition, *STARD3* protein expression was observed to be high in tissues from HER2^+^ BC, and low in TNBC patients (Figure 2E). Taken together, these results indicated that *STARD3* mRNA and protein expression levels were high in HER2^+^ BC cell lines and BC clinical samples.

Next, we examined the correlation between *STARD3* and HER2 or ER using the GEPIA and Oncomine databases. According to GEPIA, the *STARD3 *gene was strongly correlated with HER2 (P =1.4E^-92^); however, there was no correlation between *STARD3* and ER (P = 0.57) (Figure 3A). In addition, ERBB2 was the second highest gene correlated to *STARD3* after ECM1 based on the Oncomine database (Figure 3B). These results indicated that *STARD3* is highly correlated with several BC genes, particularly *HER2*.

**Prognostic significance of** ***STARD3*** **in breast cancer patients**

Based on the Kaplan-Meier survival plotter database, in BC, a high level of *STARD3* mRNA was significantly associated with worse OS (P = 9.7E^-6^\ HR = 1.67), RFS (P = 0.00029\ HR = 1.25) and DMFS (P = 6.6E^-5^ \HR=1.52) compared with a low level of *STARD3*. In addition, in BC, a high level of *STARD3* mRNA was significantly associated with poor OS in HER^+^ patients compared with a low level of *STARD3* mRNA (P = 0.019\HR = 3.95) (Figure 4, A and B). Notably, poor OS was associated with a low level of *STARD3* in ER^+^ patients in comparison with a high *STARD3* level in ER^+^ patients.

To investigate the therapeutic significance of *STARD3* in BC patients, ROC analysis was performed. As shown in Figure 4C, the area under the curve (AUC) was 0.619, which suggested *STARD3* being a good biomarker for distinguishing trastuzumab non-responders from trastuzumab responders. Patients with high levels of *STARD3* had a lower response to therapy in comparison with those with low *STARD3* levels.

## Discussion

In the current study, we examined the expression of *STARD3 *at the mRNA and protein levels in BC using conventional qualitative PCR and several bioinformatics websites, such as the Oncomine, GEPIA and Expression Atlas databases. In addition, we evaluated the impact of *STARD3* as a prognostic and diagnostic biomarker in BC.

Recently, *STARD3* has been determined to be involved in the development of several types of cancer, e.g., colorectal, prostate, and gastric cancers (21, 22). However, we found that *STARD3* has the highest expression levels in BC tissues compared with other types of cancers, such as prostate and liver cancers. Although Cai *et al*. demonstrated that the expression of *STARD3* at the mRNA level was elevated in ER and TNBC cells, as indicated by the MCF7 and MDAMB-231 cell lines (23), *STARD3* was particularly correlated with HER2, as clearly indicated in the GEPIA and Oncomine databases. Notably, *STARD3* expression decreased in ER^+^ and in ER^+^ and TNBC patients in comparison with normal cells at the mRNA and protein levels. In fact, the co-overexpression of HER2 and *STARD3* is present in nearly 25% of BC cases (24). In a recent large study by Vassilev *et al*., protein expression was investigated by immunohisto-chemistry in more than 2000 human breast tumor cases. The results showed that nearly 10% of breast tumors showed high STARD3 expression when anti-STARD3 affinity-purified antibody was used (20). They also found that *STARD3* overexpression does not occur independently from *HER2* amplification, which is concordant with our findings that mRNA expression of *STARD3* in the HER2^+^ cell lines BT474, SKBR3, and HCC1419 was significantly higher than that in the ER^+^, TNBC, and normal cell lines. Moreover, based on the RNA-seq database in figure 2B, high expression of *STARD3* was found in HER2^+^ patients in comparison with the other BC subtypes (P <0.0001). 

All the above-mentioned findings make the independent role of *STARD3* overexpression unclear in the clinical setting. In general, patients with high* STARD3 *levels had poor clinical outcomes and short OS, RFS, and DMFS in comparison with those with lower levels, which is consistent with the findings of a previous study (23). In contrast, luminal A patients showed poorer survival with low levels of *STARD3*, and exhibited better OS with high *STARD3 *levels. Further research is warranted to clarify the role played by *STARD3* in luminal A subtypes. Cai *et al*. demonstrated that *STARD3* may contribute to the poor prognosis through its effect on cell-matrix adhesion of BC cells (23). This supported the RNA interference-based study by Kao *et al*. that revealed decreased cell-cycle progression and cell proliferation on targeted knockdown of *STARD3*, reinforcing its suggested role in cancer progression (25). Vassilev *et al*. attempted to find out if there is a role of STARD3 in promoting cell survival independently of the amplification of *HER2*. They generated HER2^-^(MCF-7) cell lines overexpressing STARD3 and green fluorescent protein (GFP) or only soluble GFP as the control. Interestingly, significant morphological differences were found between the STARD3-GFP-overexpressing cells and the control cells as demonstrated by GFP-fluorescence and phase-contrast images. The STARD3-GFP-overexpressing cells showed loss of contact inhibition and adherence to substratum. More importantly, cell data showed that STARD3 overexpression promoted Src signaling as highlighted by the increased phosphorylation of focal adhesion kinase (FAK) in the absence of *HER2* amplification. All these *in vitro* findings suggest the potential role of *STARD3* overexpression in the aggressiveness of BC. One of the possible mechanisms for trastuzumab resistance is the activation of *Src* signaling pathways (26, 27).

The specific role played by STARD3 alone in BC still need further investigation. It has been reported that loss of STARD3 protein function in MCF7 cells results in decreased cell growth but not in TNBC cells, as indicated by MDA-MB-231 (23). Overexpression of STARD3 in MCF7 cells resulted in dysregulation of cholesterol homeostasis and cancer progression by enhancing the HMG CoA reductase enzyme, which is responsible for cholesterol biosynthesis (18).

Because* STARD3* is highly expressed and contributes to cancer progression in BC, it may be considered a good biomarker and therapeutic target. Recently, a* STARD3* inhibitor was developed and tested in several BC and colon cancer cell lines but not in HER2^+^ cells. The results were promising and warrant further *in vitro* and *in vivo *investigation (28).

In conclusion, we show that elevated levels of *STARD3* are strongly correlated with HER2 but not ER. In addition, a high level of *STARD3* mRNA was associated with poor clinical OS, RFS, and DFS in BC and HER2^+^ BC. Notably, a low level of *STARD3* mRNA was associated with worse clinical outcomes in ER^+^ patients. Based on our analysis, *STARD3* may represent a potential diagnostic and prognostic biomarker for HER2^+ ^BC.
